# Combining serum miRNAs, CEA, and CYFRA21-1 with imaging and clinical features to distinguish benign and malignant pulmonary nodules: a pilot study: Xianfeng Li et al.: Combining biomarker, imaging, and clinical features to distinguish pulmonary nodules

**DOI:** 10.1186/s12957-017-1171-y

**Published:** 2017-05-25

**Authors:** Xianfeng Li, Qinghua Zhang, Xiayun Jin, Lihua Cao

**Affiliations:** 1 Department of Graduate School, the Second Hospital Affiliated to Dalian Medical University, Dalian, 116027 Liaoning China; 2 Department of Clinical Medicine, the Second Hospital Affiliated to Dalian Medical University, Dalian, 116027 Liaoning China; 3 Department of Respiratory Medicine, the Second Hospital Affiliated to Dalian Medical University, No.467 Zhongshan Road, Shahekou District, Dalian, 116027 Liaoning China

**Keywords:** Pulmonary nodule, Diagnosis, Biomarker, Imaging, Lung cancer

## Abstract

**﻿﻿Background:**

﻿Our study was designed to improve the accuracy of determining whether pulmonary nodules are benign or malignant.

**Methods:**

We evaluated the clinical and imaging features and serum markers: neuron specific enolase (NSE), carcino-embryonic antigen (CEA), cytokeratin fragment antigen 21–1 (CYFRA 21–1), miRNA-21-5p, and miR-574-5pof in 39 patients with pathology information. Factors that differed significantly between those with benign versus malignant pulmonary nodules were used to establish a prediction model for identifying malignant nodules.

**Results:**

The studied nodules were 51.3% malignant and 48.7% benign. Age, smoking status, nodule diameter, history of emphysema, vascular sign, burr sign, CYFRA21-1, CEA, miRNA-21-5p, and miRNA-574-5p differed significantly between the benign and malignant nodule groups. Serum levels of CYRFA21-1 and CEA could be used to distinguish between malignant and benign nodules with a positive predictive value (PPV) of 80.0%, a negative predictive value (NPV) of 84.2%, and an area under the receiver operating characteristics curve (AUC) of 0.863. Using the serum levels of miRNA-21-5p and miRNA-574-5p, the PPV was 55%, the NPV was 84.2%, and the AUC was 0.797. When all four serum markers were combined, the PPV was 80%, the NPV was 89.5%, and the AUC was 0.921. We established a prediction model for malignant nodules, including clinical features, imaging features, and serum markers. In cross-validation, the ratio of discriminant conformance was 95%.

**Conclusions:**

Serum levels of miRNA-21-5p and miRNA-574-5p are significantly higher in patients with malignant nodules than in patients with benign nodules and are potential serum biomarkers. Our prediction model could improve malignant nodule diagnosis.

## Background

Lung cancer is the most common malignancy, causing more than 1.3 million deaths annually worldwide, including approximately 400,000 deaths in China alone [1]. Currently, the 5-year survival rate for lung cancer is only 15%, but early diagnosis allows that rate to rise to 70–80% [2, 3]. Previously, we found that lung nodules were detected in 39.1% of patients at high risk of lung cancer screened with low-dose computed tomography (CT) scanning, of which 96.4% were benign [4]. Currently, a biopsy of lung tissue obtained through bronchoscopy, CT-guided percutaneous approaches, or open surgical procedures is required to distinguish benign and malignant pulmonary nodules pathologically. However, these procedures are invasive and high risk, are costly, and sometimes patients cannot tolerate them. It is important to develop less invasive and expensive techniques to aid in monitoring patients with lung nodules for either benign conditions or early-stage cancer.

Serum microRNAs (miRNAs) are novel tumor markers that can be used to screen for cancer [5], providing a new approach for the early diagnosis of lung cancer. Lawrie et al. [6] proved that miRNA-21, miRNA-210, and miRNA-15 are expressed at high levels in the serum of patients with lung cancer, with miRNA-21 being the most prominent. Wei et al. [7] showed that plasma miRNA-21 can serve as a circulating tumor biomarker for the early diagnosis of non-small cell lung cancer, yielding an area under the receiver operating characteristic curve (AUC) of 0.775 (95% confidence interval 0.681–0.868), with 76.2% sensitivity and 70.0% specificity. Foss et al.[8] showed that miRNA-574-5p levels can be used in the early diagnosis of non-small cell lung cancer; the combination of miRNA-574-5p with miRNA-1254 provided a sensitivity and specificity of 82 and 77%, respectively. Few studies have compared miRNAs with conventional markers like neuron-specific enolase (NSE), carcinoembryonic antigen (CEA), and cytokeratin fragment antigen 21–1 (CYFRA21-1), and little has been published regarding the use of miRNAs in the early diagnosis of benign and malignant pulmonary nodules. Detection of multiple tumor markers, screening the best combination of miRNAs, and comprehensive analysis of biomarkers and imaging are a growing current trend for noninvasive detection of lung cancer.

Currently, clinical experts evaluate pulmonary nodules through common methods, such as detection of serum tumor markers, the pulmonary nodules reviewer’s guide developed by the American College of Chest Physicians [9], Swensen’s model [10], the VA model [11], and other methods [12, 13]. However, the sensitivity and specificity of these methods for discriminating benign versus malignant pulmonary nodules are not optimal. Our study was designed to explore the value of miRNAs as serum tumor markers in distinguishing benign from malignant pulmonary nodules, and to compare miRNAs with conventional markers like CEA, NSE, and CYRFA21-1. We sought to establish a prediction model of malignant nodules by combining clinical features, imaging features, and serum tumor markers that may improve the accuracy of distinguishing benign from malignant pulmonary nodules.

## Methods

### Clinical and imaging features

We recorded the clinical features and imaging features of 39 patients in whom pulmonary nodules had been discovered initially from March 2015 to November 2015 in the Second Affiliated Hospital of Dalian Medical University. Inclusion criteria were age 20–80 years, nodule diameter between 5 and 30 mm, and at least one pulmonary nodule identified by chest CT. We excluded patients with severe complications. Before surgery and drug treatment, we collected peripheral blood samples and centrifuged them to obtain serum samples, which were stored at −80 °C. Clinical characteristics included age, sex, smoking history, family history of cancer, and personal history of emphysema. The imaging features of pulmonary nodules included nodules position (i.e., lingula, left lower lobe, right upper lobe, or right lower lobe), diameter, density (solid, mixed, or hyaline membrane), edge features (smooth, lobulated, or irregular), calcification, tracheal signs, vascular signs, vacuole signs, and the pleural pull sign. Clinical radiologists blinded to the clinical information of the patients and the final pathology results of the nodules assessed the imaging features of nodules. Pathological findings were available for all 39 nodules, either through surgery or biopsies performed via bronchoscopy. All patients had early-stage lesions.

### The detection of CEA, NSE, and CYFRA21-1

The serum levels of tumor markers CEA, NSE, and CYFRA21-1 were measured with an ELISA kit (Phoenix Pharmaceuticals, INC, USA) following the manufacturer’s instructions. The same diagnostic kits were used for each tumor marker.

### The detection of miRNA-21-5p and miRNA-574-5p

Stored serum samples were thawed, and 300 μl was added to 250 fmol of the standard cel-miRNA-39 30 μl in 900 μl Trizol reagent (Invitrogen, San Diego, CA, USA). Total RNA was extracted according to the manufacturer’s instructions. For quantitative, real-time PCR for miRNA-21-5p, miRNA-574-5p, and cel-miRNA-39, miRNA-specific TaqMan MicroRNA Assays (Applied Biosystems, Foster City, CA, USA) were used. The specific stem-loop reverse transcription primers for miRNA-21-5p and miRNA-574-5p were provided by TAKARA Biotechnology (miRNA-21-5p: 5′-UAG CUU AUCAGACU GAUGUUGA-3′; miRNA-574-5p: 5′-UGAGUGUGUGUGUGU GAG UGUG U-3′). The total reaction volume for reverse transcription was 20 μl and included total RNA, 50 ng–5 μg; GSP (gene-specific primers), 2 pmol; 2× ES Reaction Mix, 10 μl; Easy Script RT/RI Enzyme Mix, 1 μl; and sufficient RNase-free water to a final volume of 20 μl. The reverse transcription reaction proceeded at 42 °C for 15 min, followed by heat inactivation at 85 °C for 5 s. The real-time PCR reaction also used a volume of 20 μl, assayed in triplicate for each sample, and included cDNA, 3 μl; forward primer (10 μM), 0.4 μl; reverse primer (10 μM), 0.4 μl; 2× TransStart Top Green qPCR SuperMix, 10 μl; passive reference dye (50×) (optional), 0.4 μl; and sufficient distilled, deionized water to a final volume of 20 μl. The reactions were performed in an ABI Prism 7500 qPCR machine, and the reaction conditions were 94 °C denaturation for 35 s, 60 °C annealing for 34 s, 72 °C extension for 10 s, with 40 cycles of the reaction.

The target gene to be detected was normalized with nematode cel-miRNA-39 exogenous internal control. miRNA expression levels were calculated by the △△Ct method: △Ct = mean value Ct (miRNA of interest) − mean value Ct (reference miR-39), in this case △△Ct = mean value Ct (miRNA of malignant nodules) − mean value Ct (miRNA of benign nodules). The relative expression of each miRNA of interest corresponded to the 2^△△Ct^ value [7].

### Statistical analyses

Statistical analyses were performed with SPSS 17.0 and Graphpad Prism 5.01. The test for normal distribution used the Kolmogorov-Smirnov test. The Wilcoxon rank-sum test was used to analyze data that were not normally distributed. When the data was normally distributed, we used a *t* test for univariate analysis, while for classification data we used Fisher’s exact test, and for multiple sets of measurement data, we used variance analysis. *P* values <0.05 were considered significant. We selected index differences between the two groups using SPSS 17.0 statistical software. We used Fisher discriminant analysis to establish a prediction model for malignant nodules.

## Results

### Clinical features help differentiate benign and malignant nodules

For the 39 patients with pulmonary nodules included in this study, pathology results were available from material obtained by surgery or bronchoscopic biopsy. There were 20 (51.3%) malignant nodules, of which 6 were squamous cell carcinoma, 13 were adenocarcinoma, and 1 was a neuroendocrine carcinoma. The remaining 19 (48.7%) were benign nodules, of which 4 were hamartoma, 3 were tuberculosis granulomas, 4 were inflammatory lesions, 1 was lymph node hyperplasia, 1 was a granulomatous lesion, 1 was sarcoidosis, and 5 were other benign tumors.

Patient clinical data differed between patients with benign vs. malignant nodules (Table 1). The average ages of those with benign and malignant nodules were 53.11 ± 11.66 years and 63.25 ± 9.48 years, respectively (*P* = 0.005). For smoking, 26.32% of those with benign nodules were smokers, with 64.21 ± 131.42 cigarettes/year, while 70% of those with malignant nodules were smokers, with 565.00 ± 499.76 cigarettes/year (*P* = 0.01 for smoking history and 0.001 for smoking). Emphysema was present in 25% of those with malignant nodules, while none of those with benign nodules had emphysema (*P* = 0.04). Sex and family history of cancer did not differ significantly between the groups.

**Table 1:** Comparison of clinical features and imaging features between benign and malignant nodules

Characteristic	Benign lung nodules group (*n* = 19)	Malignant lung nodules group (*n* = 20)	*P* value
Clinical			
Age, years	53.11 ± 11.66	63.25 ± 9.48	0.005^a^
Male, %	47.37	75.00	0.12^c^
Smoking history, %	26.32	70.00	0.01^c^
Smoking, cigarettes/year	64.21 ± 131.42	565.00 ± 499.76	0.001^a^
Family history of cancer, %	5.26	15.00	0.61^c^
Emphysema, %	0.00	25.00	0.04^c^
Radiological			
Location, %			
LUL	26.32	25.00	
LLL	15.79	0	
RUL	42.10	30.00	
RLL	15.79	45.00	0.10^b^
Diameter, mm	15.21 ± 6.98	21.50 ± 7.06	<0.001^a^
Density, %			
Solid	78.95	65.00	
Hyaline membrane	10.53	20.00	
Mixed	10.53	15.00	0.61^b^
Lobulation, %	10.53	30.00	0.24^c^
Spiculation, %	31.58	70.00	0.03^c^
Air bronchogram, %	21.05	20.00	1.00^c^
Vessels sign, %	0.00	30.00	0.02^c^
Cavitation, %	0.00	20.00	0.11^c^
Calcification, %	15.79	0.00	0.11^c^
Pleural tail, %	31.58	55.00	0.20^c^

Data are *N* (%) or mean ± SD. *P <* 0.05 was considered statistically significant

*LUL* left upper lobe, *LLL* left lower lobe, *RUL* right upper lobe, *RLL* right lower lobe

^a^ *t* test

^b^chi-square test

^c^Fisher exact test

### Imaging characteristics help differentiate benign and malignant pulmonary nodules

We analyzed the imaging feature of pulmonary nodules, including the location, diameter, density, edge characteristics, calcifications, tracheal signs, vascular signs, vacuole signs, and presence of pleural traction. Benign nodules were significantly smaller than malignant ones (15.21 ± 6.98 mm vs. 21.50 ± 7.06 mm, *P* < 0.001). Spiculation was present in 31.58% of benign nodules and 70% of malignant nodules (*P* = 0.03), while a vascular sign was present in 0.00% and 30%, respectively (*P* = 0.02). Other imaging features, including location, density, lobulation, vacuole sign, and pleural traction, did not differ significantly between the groups (Table 1).

### The levels of serum miRNA-21-5p and miRNA-574-5p in patients with malignant nodules were significantly higher than in those with benign nodules

We measured the relative expression levels of serum miRNAs by real-time PCR. The levels of miRNA-21-5p were 1.99 ± 2.80 and 6.63 ± 14.39, respectively, between the benign and malignant nodules groups (*P* = 0.02), while the levels of miRNA-574-5p were 2.59 ± 2.92 and 86.75 ± 288.25, respectively (*P* = 0.047) (Table 2).

**Table 2:** Comparison of serum tumor marker levels between benign and malignant nodules

Serum tumor marker	Benign lung nodules group (*n* = 19)	Malignant lung nodules group (*n* = 20)	*P* value
CYFRA21-1 (ng/ml)	1.90 ± 0.77	4.39 ± 4.91	<0.001
NSE (ng/ml)	17.30 ± 4.39	15.95 ± 4.02	0.324
CEA (ng/ml)	1.08 ± 0.68	2.37 ± 1.97	0.026
miRNA-21-5p	1.99 ± 2.80	6.63 ± 14.40	0.002
miRNA-574-5p	2.59 ± 2.92	86.75 ± 288.25	0.047

Date are presented as means ± SDs. *P <* 0.05 was considered statistically significant

### Combining of the levels of serum miRNA-21-5p, miRNA-574-5p, CEA, and CYRFA21-1 can improve the accuracy of diagnosis of malignant nodules

**Fig. 1:**
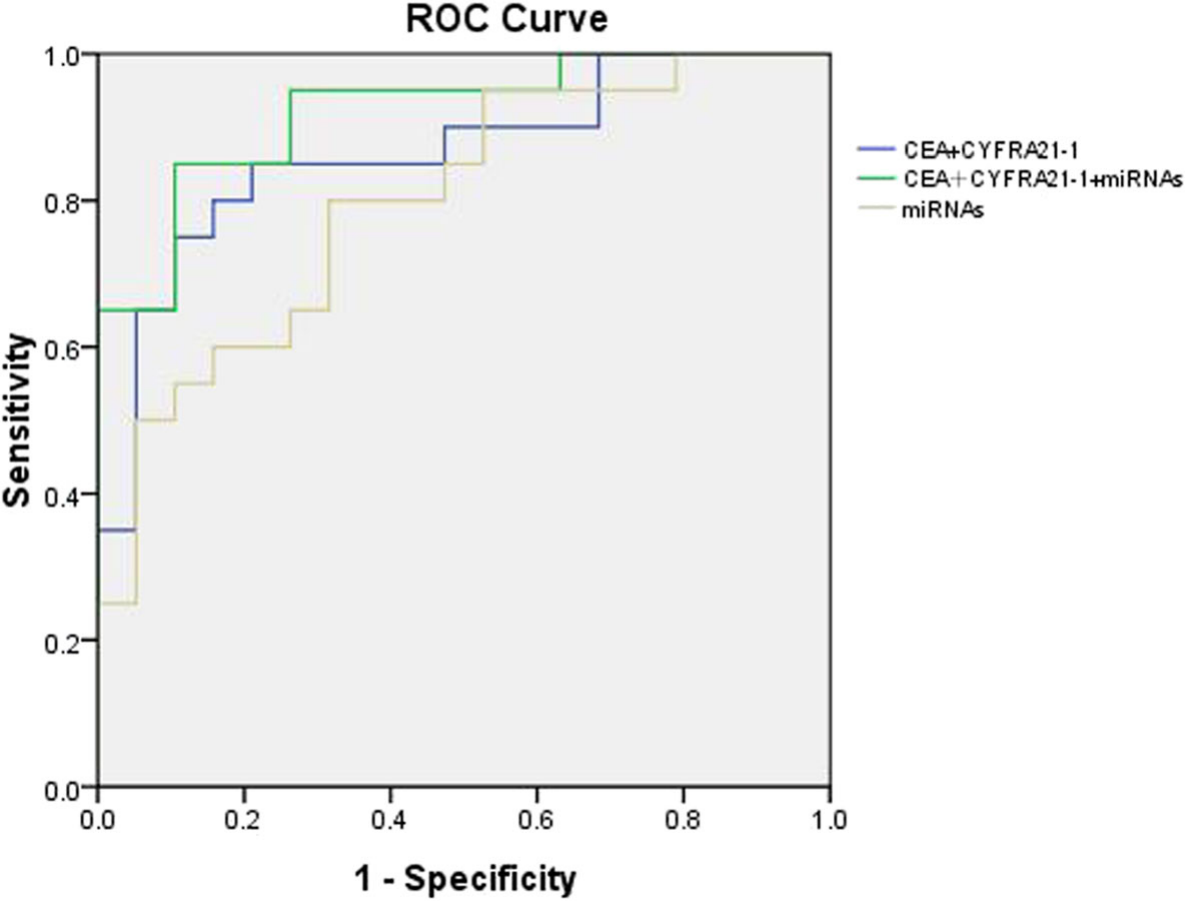
ROC curve analysis of serum factors for predicting malignancy of pulmonary nodules. When serum markers CEA and CYFRA21-1 were used, the AUC was 0.863. When the levels of serum miRNA-21-5p and miRNA-574-5p were used, the AUC was 0.797. When all four serum markers (CEA, CYFRA21-1, mRNA-21-5p, and miRNA-574-5p) were used, the AUC was 0.921

We measured the levels of serum NSE, CEA, and CYRFA21-1 in patients with pulmonary nodules. The levels of CYFRA21-1 were 1.90 ± 0.77 ng/ml in the benign nodules group and 4.39 ± 4.91 ng/ml in the malignant nodules group (*P* < 0.001) while the CEA levels were 1.08 ± 0.68 ng/ml and 2.37 ± 1.97 ng/ml, respectively (*P* = 0.026). NSE levels did not differ significantly between the groups. When combining serum markers CYRFA21-1 and CEA, the positive predictive value (PPV) for malignant pulmonary nodules was 80.0%, the negative predictive value (NPV) was 84.2%, and the AUC was 0.863. When combining miRNA-21-5p and miRNA-574-5p, the PPV for malignant pulmonary nodules was 55%, the NPV was 84.2%, and the AUC was 0.797. Combining the four serum markers, CEA, CYRFA21-1, miRNA-21-5p, and miRNA-574-5p, the PPV for malignant pulmonary nodules was 80%, the NPV was 89.5%, and the AUC was 0.921. Therefore, combining the four serum markers could improve the accuracy of identification of benign and malignant nodules (Fig. 1).

### Establishing a prediction model of malignant pulmonary nodules by combining the clinical features, imaging features, and serum tumor markers to improve the accuracy of diagnosis of pulmonary nodules

We selected the factors that differed significantly between benign and malignant pulmonary nodules, such as age, smoking, emphysema, diameter, spiculation (burr), vascular sign, and the serum levels of CYRFA21-1, CEA, miRNA-21-5p, and miR-574-5p to establish a prediction model for malignant nodules. We applied Fisher discriminant analysis. (1) *Y* = 0.275 × age + 0.704 × smoking + 0.29 × diameter + 0.38 × emphysema + 0.891 × vascular sign + 0.481 × burr + 2.775 × (CYFRA21-1) + 0.061 × CEA + 0.304 × (miRNA-21-5p) − 2.778 × (miRNA-574-5p); (*Y* > 0 ruled malignant; *Y* < 0 ruled benign). According to the cross-validation, the ratio of discriminant conformance was 95%, indicating that the discriminant effect of this model is very good. (2) Estimating the probability formula *P* = exp (*Y*_1_-*Y*max)/exp (*Y*_1_-*Y*max) + exp (*Y*_2_-*Y*max). (3) exp = e (*Y*_1_-*Y*max)/e (*Y*_1_-*Y*max) + e (*Y*_2_-*Y*max). (4) *Y*_1_ = 0.546 × age + 0.007 × smoking + 0.58 × diameter + 22.899 × emphysema + 27.887 × vascular sign + 8.628 × burr + 5.162 × (CYFRA21-1) −0.624 × CEA + 0.186 × (miRNA-21-5p) − 0.098 × (miRNA-574-5p) − 55.555. (5) *Y*_2_ = 0.649 × age + 0.015 × smoking + 0.744 × nodule size + 27.613 × emphysema + 38.335 × vascular sign + 12.643 × burr + 8.244 × (CYFRA21-1) − 0.464 × CEA + 0.301 × (miRNA-21-5p) − 0.151 × (miRNA-574-5p) − 98.349. *Y*max is the larger of *Y*1 and *Y*2. Our study added the new serum tumor markers miRNA-21-5p and miRNA-574-5p, which have improved the accuracy of identification of benign and malignant nodules.

## Discussion

With the popularity of low-dose chest CT, the detection rate of lung nodules has risen dramatically, though most of these nodules are benign. To achieve early diagnosis of malignant nodules and avoid invasive evaluation of benign nodules, clinicians need to discriminate accurately between benign and malignant nodules. In clinical practice, clinicians use various methods to diagnose or follow pulmonary nodules. Firstly, they may follow the patients with lung nodules to monitor the nodules for changes. Alternatively, one may need to obtain a pathological diagnosis by transbronchial lung biopsy or surgical biopsy, particularly for those nodules that are likely to be malignant. In making these decisions, the clinical approach needs to consider the comorbidities of the patient as well as the patient’s wishes [14].

With increasing knowledge about the imaging characteristics and serum tumor biomarkers of pulmonary nodules, there is great impetus to find a non-invasive method with higher specificity and sensitivity than can be obtained using current imaging models or biomarkers, and to achieve reliable early diagnosis of pulmonary malignancy. Our study aimed to find more effective new tumor markers and to establish a predictive model with serum tumor markers, as well as clinical features and imaging features, to improve the rate of early diagnosis of lung cancer.

For radiological findings, clinicians use the Swensen model [10] and the VA model [11] commonly to distinguish between benign and malignant lung nodules. In the Swensen model, factors such as age, smoking history, history of other cancers, diameter, spiculation, and location of the nodules have important significance in judging between benign and malignant nodules [15]. We found that age, smoking history, emphysema, diameter, spiculation, and vascular sign have statistical significance in differentiating benign and malignant nodules. Malignant nodules are more likely in patients who are older and who have a longer smoking history. We also found that nodules are more likely to be malignant when spiculation (burr sign) and a vascular sign are present. These results are consistent with previous reports.

Our results showed that the location of lung nodules had no significant power to discriminate between benign and malignant nodules, perhaps because of the small sample size of this experiment. We will expand the sample size in the future to assess the significance of the location in the differential diagnosis of pulmonary nodules. However, the specificity and sensitivity is not satisfactory when using only clinical and imaging information to determine whether nodules are benign or malignant.

We were able to add serum tumor markers to clinical and imaging features to establish a prediction model for malignant nodules, improving the accuracy of discriminating pulmonary nodules. NSE, CEA, and CYFRA21-1 as serum tumor markers have been used routinely in clinical testing, but their specificity is not high.

Serum miRNAs are novel serum tumor markers that are non-invasive, easily obtained, and have high sensitivity. It has been confirmed that miRNAs are involved in the development of a variety of tumors, such as neuroblastoma [16], pituitary adenoma [17], leukemia [18], thyroid cancer [19], hepatocellular carcinoma [20], breast cancer [21], and lung cancer [22]. miRNA-21 is an especially important miRNA molecule of more than 700 different miRNAs, as it is highly expressed in breast cancer, colon cancer, lung cancer, and other tumors [23–25]. The study of Foss et al.[8] showed that miRNA-574-5p has important significance in the early diagnosis of non-small cell lung cancer. When miRNA-574-5p and miRNA-1254 were used together, the sensitivity and specificity were 82 and 77%, respectively, for early diagnosis of lung cancer.

Our experiments attempted to verify these miRNAs for early identification of pulmonary nodules, and to explore the significance of combining them with other factors. We found that miRNA-21-5p and miRNA-574- 5p were highly expressed in the serum of patients with malignant pulmonary nodules, confirming the important role of miRNAs for screening early malignant lung nodules. miRNA-21-5p and miRNA-574-5p are potential new serum markers for lung cancer. In our study, the combination of miRNA-21-5p and miRNA-574-5p had a PPV for identifying malignant lung nodules of 55%, an NPV of 84.2%, and an AUC of 0.797. The serum markers CYRFA21-1 and CEA had a PPV of 80.0%, an NPV of 84.2%, and an AUC of 0.863. When we combined all four serum markers, CEA, CYRFA21-1, miRNA-21-5p, and miRNA-574-5p, the PPV was 80%, the NPV was 89.5%, and the AUC was 0.921. Therefore, the combined serum markers CYRFA21-1, CEA, miRNA-21-5p, and miRNA-574-5p can significantly improve the accuracy of distinguishing benign and malignant pulmonary nodules.

The American College of Chest Physicians guidelines on pulmonary nodule evaluation recommend the use of models to predict the probability of malignancy [9]. As these guidelines recommend, one should assess the probability of malignancy of any nodules. In this context, we offer the following clinical model using comprehensive available clinical information and describe our calculation method. In this study, we have analyzed the clinical features, imaging characteristics, and serum tumor markers of patients with newly discovered primary pulmonary nodules. We then selected the factors of age, smoking history, emphysema, diameter, spiculation, vascular sign, and serum CEA, CYFRA21-1, miRNA-21-5p, and miRNA-574-5p levels, all of which were significantly different between benign and malignant nodules. These factors were used to establish a predictive model for malignant nodules. Compared with previous prediction models, our model added the conventional tumor markers CEA and CYFRA21-1 and serum miRNA-21-5p and miRNA-574-5p as risk factors, which achieved a good discriminant result. However, this is a preliminary study and still requires further study and validation. At present, the model has not been externally verified. We will validate the model in the future and compare the model with the classical VA and Swensen models.

Our preliminary study discussed the role of miRNA-21-5p and miRNA-574-5p in the diagnosis of pulmonary nodules. It shows that combining multiple tumor markers with imaging features may enable clinicians to identify malignant pulmonary nodules earlier. However, this study has a relatively small sample size, and the specimen collection has geographic limitations. The model lacks a longitudinal comparison with other clinical models. miRNAs as novel serum tumor markers are important for the early diagnosis of malignant pulmonary nodules, but the detection method thus far has limited clinical application. Therefore, a large-scale clinical study is required to verify the importance and utility of these markers. We will verify the accuracy of the model through further research by expanding the sample size, collecting more patients with pulmonary nodules from different regions, and extending the follow-up time. Finding a new treatment target for lung cancer is the next focus of our work. We will improve the rate of survival and prognosis of patients through earlier diagnosis and treatment of lung cancer.

## Conclusions

Serum levels of miRNA-21-5p and miRNA-574-5p are significantly higher in patients with malignant nodules than in patients with benign nodules, and thus can be considered potential serum biomarkers. Combining serum miRNA-21-5p, miRNA-574-5p, CYFRA21-1, and CEA data could improve malignant nodule prediction accuracy. Our prediction model, which encompasses clinical features, imaging features, and serum tumor markers, could improve malignant nodule diagnosis. Our study, including the model we established, are thus important for the clinical identification of benign and malignant pulmonary nodules.

## Abbreviations

NSE: Neuron-specific enolase
CEA: Carcinoembryonic antigen
CYFRA21-1: Cytokeratin fragmet angtigen 21–1
PPV: Positive predictive value
NPV: Negative predictive value
AUC: Area under the receiver operating characteristics curve
CT: Computed tomography
